# Digitally Tunable Dispersion Controller Using Low‐Loss Silicon Nitride Multimode Waveguide Gratings

**DOI:** 10.1002/nap2.70072

**Published:** 2026-04-15

**Authors:** Weihan Wang, Ruitao Ma, Shujun Liu, Mingyu Zhu, Zian Cao, Zexu Wang, Weike Zhao, Yiwei Xie, Zejie Yu, Daoxin Dai

**Affiliations:** ^1^ State Key Laboratory of Extreme Photonics and Instrumentation, Center for Optical & Electromagnetic Research, College of Optical Science and Engineering, International Research Center for Advanced Photonics Zhejiang University Hangzhou China; ^2^ China Jiliang University Hangzhou China; ^3^ Intelligent Optics & Photonics Research Center Jiaxing Research Institute Zhejiang University Jiaxing China

**Keywords:** chirped multimode waveguide gratings, digital tuning, dispersion tuning, silicon nitride

## Abstract

A digitally tunable dispersion controller (DTDC) using low‐loss silicon nitride multimode waveguide gratings is developed. The architecture of the DTDC is based on *N* stages of cascaded chirped multimode waveguide gratings (CMWGs) and (*N* + 1) 2 × 2 Mach–Zehnder switches (MZSs). The *n*th stage incorporates *m* = 2^*n*−1^ CMWGs, resulting in a total of *M* = 2^*N*^ − 1 CMWGs across the entire structure. The overall dispersion is determined by the collective states of all the MZSs. The DTDC enables a digital tuning range of dispersion from 0 to (2^*N*^ − 1) *D*_0_ with a minimum step of *D*_0_, where *D*_0_ is defined as the dispersion provided by a single CMWG. A DTDC designed with *N* = 5 and *M* = 31 is fabricated with 400‐nm‐thick silicon nitride via a standardized 8‐inch foundry process, providing an average dispersion *D*_0_ of approximately 1.60 ps/nm per single 4‐mm‐long CMWG over a 26‐nm‐wide bandwidth. The total dispersion can be digitally tuned from 0 to 49.72 ps/nm by controlling the MZSs appropriately, and the corresponding group delay can be tuned from 0 to 1304 ps. The group delay ripple (GDR) accounts for as low as 1.14% of the total delay, with the propagation loss estimated to be 0.22 dB/cm.

## Graphical abstract

A digitally tunable dispersion controller (DTDC) using low‐loss silicon nitride multimode waveguide gratings is demonstrated. Utilizing cascaded chirped multimode waveguide gratings and Mach–Zehnder switches, the device achieves a tuning range of 0–49.72 ps/nm with 1.60 ps/nm steps over a 26‐nm bandwidth. The structure features low propagation loss (0.22 dB/cm) and minimal group delay ripple (1.14% of total delay).

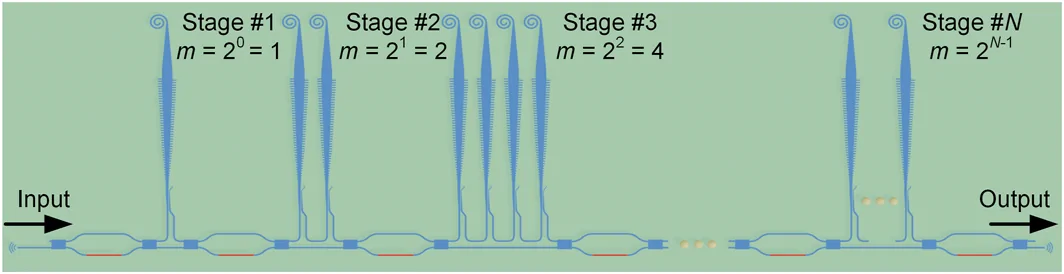

## 1 Introduction

Chromatic dispersion, which arises from the varying group velocities of different wavelengths, is a fundamental and critical phenomenon in numerous optical systems, including microwave photonics [1, 2], ultrafast pulse manipulation [3], and high‐speed long‐haul optical communication [4]. For instance, a spectral shaper based on a dispersion controller is combined with time‐wavelength mapping to generate a linearly chirped microwave [5]. In time‐lens systems, an initial flat pulse with broadband spectrum is produced by cascaded amplitude modulator and phase modulator, and precise dispersion management is then conducted to achieve femtosecond pulse generation [6]. When it comes to ultrafast pulse amplification, dispersion management is typically employed to broaden the initial pulse to reduce the undesired nonlinear effects at high peak power and subsequently is used to conduct recompression after amplification [7]. In high‐speed and long‐haul optical communication systems, the accumulated relative delay between carriers and sidebands will induce signal distortion and dispersion management is essential to improve signal integrity [8]. Besides, dispersion management also plays a vital role in arbitrary waveform generation [9], beamforming [10], pulse compression [11, 12], spectrum compression [13, 14, 15], supercontinuum generation [16], and soliton generation [17].

To date, various on‐chip structures have been explored for dispersion management, including micro‐ring resonators (MRRs) [18], arrayed waveguide gratings (AWGs) [19], Mach–Zehnder interferometers (MZIs) [20], contra‐directional couplers (contra‐DCs) [21], and waveguide Bragg gratings [22]. However, each approach is constrained by distinct limitations that impede their widespread deployment. MRRs, for instance, typically exhibit a severely limited operation bandwidth and require active thermal stabilization, introducing extra complexity and power consumption. MZIs suffer from a narrow bandwidth and large footprints, which diminish their suitability for compact integration. Contra‐DCs, composed of two strip waveguides, afford limited design flexibility and introduce additional fabrication complexity. Singlemode waveguide gratings are inherently limited by the difficulty of separating the back‐reflected signals from the input port, which necessitates the use of external circulators and thus hinders large‐scale on‐chip integration [22]. Efforts have been made to explore circulator‐free configurations, such as integrating the grating with a 3‐dB Y‐splitter, which however introduces an intrinsic insertion loss of 6 dB [6]. Alternatively, employing a 2 × 2 multimode interferometer (MMI) combined with paired gratings can achieve signal separation, yet it doubles the device footprint and demands stringent structural consistency [23, 24].

Recently, multimode waveguide gratings have attracted significant interest due to their circulator‐free characteristic and scalability [25, 26, 27, 28]. By converting the TE_0_ mode into the TE_1_ mode, the input and back‐reflected signals can be spatially separated via an efficient mode (de)multiplexer. Early demonstration on the silicon‐on‐insulator (SOI) platform consists of 15 2‐mm‐long chirped multimode waveguide gratings (CMWGs) integrated with five Mach–Zehnder switches (MZSs) to achieve digital dispersion tunability [25]. A tuning range of 0–42.8 ps/nm with a step of 2.82 ps/nm over a 20‐nm‐wide bandwidth was achieved, providing a maximum group delay of 760 ps with a propagation loss of 1.3 dB/cm [25]. Another implementation was achieved with a dispersion of 6.49 ps/nm and a total group delay of 150 ps within a 22‐nm‐wide bandwidth by using an Archimedean spiral waveguide, while the total footprint is 0.21 × 0.21 mm^2^ and the propagation loss is 5 dB/cm [26]. One should note that these SOI CMWGs have a relatively high propagation loss due to the ultra‐high index contrast, limiting the potential for further scaling up. Furthermore, the ultra‐high index contrast also introduces strong dependence on the random fabrication‐induced variations, and the reflection spectra of the SOI CMWGs often have random ripples. Consequently, noticeable group delay ripples (GDRs) are introduced. Besides, the use of electron‐beam lithography (EBL) in these works tends to introduce negative impact due to the writing‐field stitching effects, posing the challenges for practical large‐scale applications if higher‐precision fabrication processes are not available yet.

Alternatively, silicon nitride has emerged as a highly promising photonic platform, due to its advantageous properties such as low propagation loss, moderate index contrast, and high thermal stability [29, 30]. Leveraging these advantages, recent research has demonstrated silicon nitride CMWGs with an Archimedean spiral, and a 20.11‐cm long device was developed to achieve a large group delay of 2864 ps [27]. However, reconfigurable and tunable dispersion control on the silicon nitride platform remains yet to be realized, which is essential for flexible signal processing in dynamic optical networks.

In this work, we develop a digitally tunable dispersion controller (DTDC) using low‐loss silicon nitride multimode waveguide gratings via a standardized 8‐inch foundry process compatible with high‐volume manufacturing for the first time. As an example, a 5‐stage DTDC with 31 4‐mm‐long CMWGs is realized on a 400‐nm‐thick silicon nitride platform. Compared to the previous SOI implementation [25], the present work significantly improves the performance of digitally tunable dispersion owing to the lowered index contrast of silicon nitride photonic waveguides used here. In particular, the propagation loss is significantly lowered to 0.22 dB/cm, which enables large‐scale photonic integration to achieve a large dispersion‐tuning range of 0–49.72 ps/nm in a broad operational bandwidth of 26 nm. With the present silicon nitride DTDC, a fine tuning step of 1.60 ps/nm and a maximal group delay up to 1304 ps is achieved in experiments. For the present silicon nitride photonic chip fabricated with foundry‐compatible DUV lithography processes, the fabrication‐induced random phase errors are lowered greatly (partially due to the lowered index contrast), leading to a substantial reduction in GDR, which is as low as 1.14% of the total delay. In addition, the present silicon nitride chip has higher thermal stability due to the lower thermo‐optic coefficient than the silicon one and also shows high cost‐effectiveness due to the wafer‐scale fabrication convenience, paving a robust scalable routine for practical applications.

## 2 Structure and Design

The detailed architecture of the proposed DTDC is presented in Figure 1a. The controller comprises *N* stages of cascaded CMWGs and (*N* + 1) 2 × 2 MZSs. The *n*th stage incorporates *m* = 2*^n^* ^−1^ CMWGs to enable digital tunability. To ensure design simplicity and high scalability, all the CMWGs utilized are identical. Each CMWG contributes a certain amount of dispersion that originates from the wavelength‐dependent propagation distance. The total dispersion *D* is accumulated by all the stages depending on the states of all the (*N* + 1) 2 × 2 MZSs, which has *D = PD*_0_, where *D*_0_ denotes the dispersion of a single CMWG. *P* denotes the number of CMWGs passed through, as given by $P=\sum_{n=1}^{N}\Gamma_{n}2^{n-1}$, where Γ_*n*_ represents the state of the MZS for the *n*th stage (Γ_*n*_ = 0 for OFF and Γ_*n*_ = 1 for ON).

**FIGURE 1:**
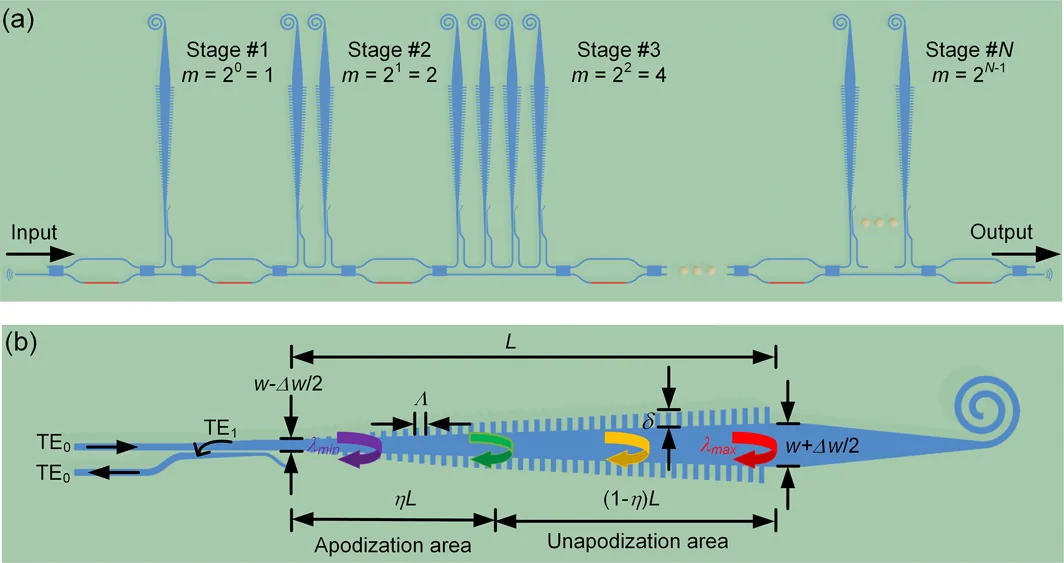
Schematic diagram. (a) Configurations of the proposed digitally tunable dispersion controller; (b) details of the integration of the CMWG and a mode (de)multiplexer.

Figure 1b illustrates more details of the CMWG and the mode (de)multiplexer. The CMWG is purposefully designed with asymmetric teeth, enabling the forward TE_0_ mode to be converted into the TE_1_ mode reflected backward. The mode (de)multiplexer is used to drop the reflected TE_1_ mode to TE_0_ mode at the output port without the need for a circulator, which is highly detrimental for integration and scalability. The width of the CMWG is gradually varied to achieve a spatially dependent effective index along the propagation direction, ensuring incident light at different wavelengths is reflected at distinct positions, in accordance with the Bragg condition: $\lambda =\left(n_{\text{eff}0}+n_{\text{eff}1}\right)\Lambda$, where *λ* denotes the reflected wavelength, Λ denotes the grating period, *n*_eff0_ denotes the effective index of the TE_0_ mode, and *n*_eff1_ denotes the effective index of the TE_1_ mode. Here, the grating period is fixed and the waveguide width gradually increases along the propagation direction to gain positive dispersion, such that longer‐wavelength components are reflected after propagating a longer distance, which means a longer time delay. Furthermore, negative dispersion can be gained by reversing the width variation trend easily if required.

Here, a silicon nitride‐on‐insulator wafer with a 400‐nm‐thick silicon nitride core and a buried‐oxide layer is used. The center width *w* of the waveguide is chosen as 1.7 μm and the grating period Λ is fixed at 480 nm to ensure the central wavelength is around 1550 nm. The length *L* of a single CMWG, which determines the total delay, is set to 4 mm and the grating duty cycle is set as 0.5. Furthermore, the transfer matrix method (TMM) is adopted to efficiently simulate the influence of the grating teeth type, the width variation Δ*w*, the corrugation depth *δ*_0_, and the apodization length ratio *η*. Figure 2a,b shows the simulation results of transmission spectra and group delay responses with rectangular, triangular and sine teeth. It is observed that the CMWG with rectangular teeth exhibits a slightly wider bandwidth and larger group delay. Conversely, both the CMWGs with triangular and sinusoidal teeth exhibit slightly higher loss near the end edges of the reflection band. This detrimental effect would be significantly amplified upon multi‐stage cascading, so the rectangular teeth are selected for the grating. Figure 2c,d shows the simulation results with different width variations Δ*w* of 200, 400 and 600 nm, which represents a trade‐off of bandwidth and dispersion value. When the width variation Δ*w* is increased from 200 to 600 nm, the dispersion decreases from 3.50 to 1.14 ps/nm, whereas the bandwidth increases from 14.5 to 40.8 nm. As it can be seen, a broader wavelength range is achievable by increasing the width variations, however it necessitates a compromise on the dispersion magnitude for a fixed device length. To achieve a balance between the target dispersion value and the operational bandwidth, the width variation Δ*w* is ultimately set to 400 nm. Figure 2e,f shows the simulation results with different corrugation depths *δ*_0_ of 150, 250 and 350 nm, which should be as weak as possible to reduce GDR while maintaining adequate reflection to minimize loss. It is evident that when the corrugation depth *δ*_0_ is set to 250 and 350 nm, signals within the operational bandwidth achieve nearly 100% reflection. Conversely, a depth of 150 nm exhibits insufficient reflectivity, a deficit which would significantly increase the total loss upon multi‐stage cascading. Thus, the corrugation depth *δ*_0_ is set as 250 nm. The GDR mainly arises from the asymmetric Fabry–Perot cavities caused by the index transition between the input straight waveguide and the grating corrugations. Apodization technology is usually used to address this issue. Here, cosine apodization is implemented, modulating the grating corrugation according to the function given by

(1) $$\left\{\begin{array}{ll} \delta =\delta_{0}\;\sin\left(\frac{\pi l}{2\eta L}\right), & 0\leq l\leq \eta L\\ \delta =\delta_{0}, & \eta L<l\leq L \end{array}\right..$$

**FIGURE 2:**
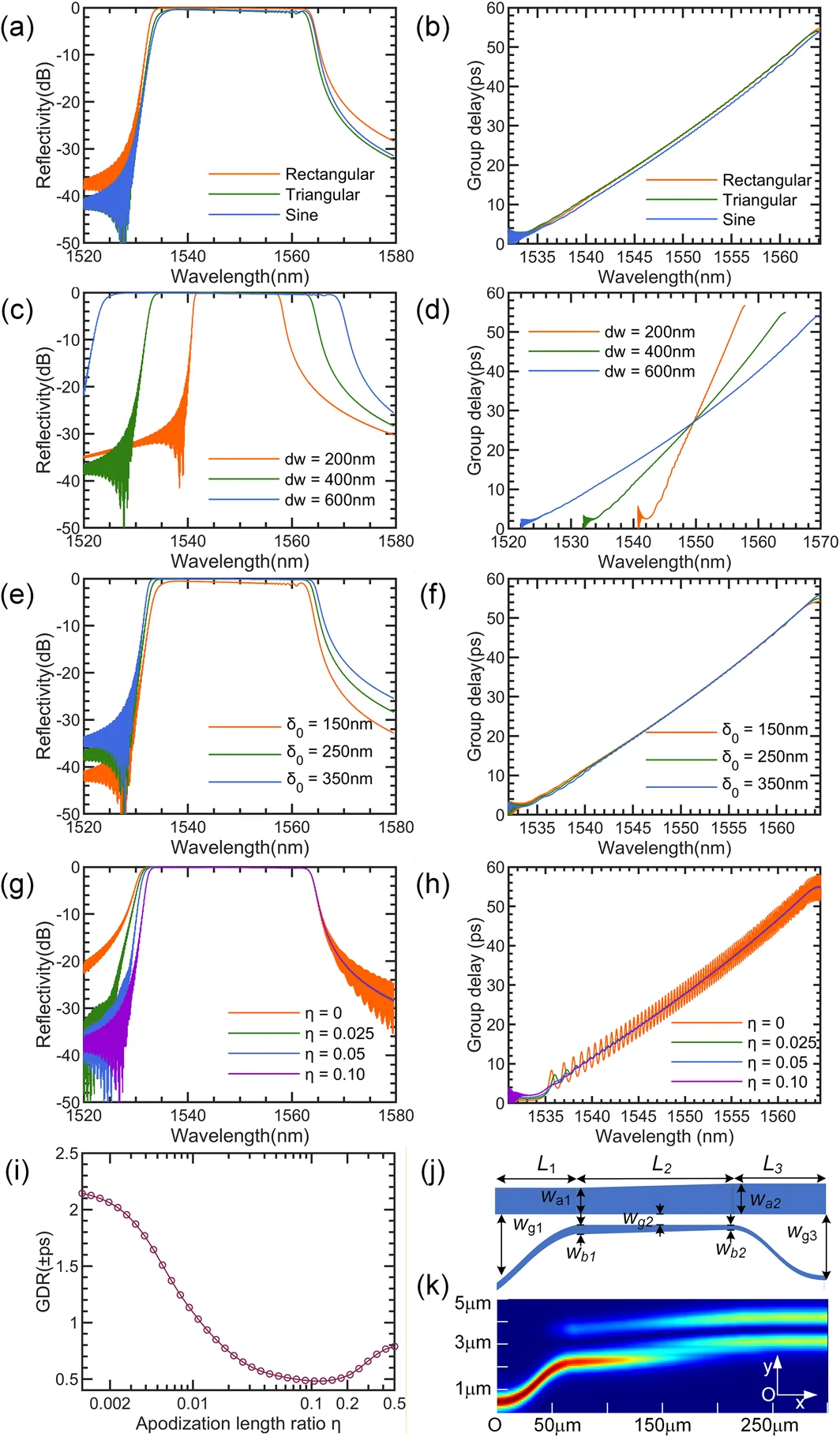
(a, b) Simulation results of the transmission spectra (a) and group delay responses (b) for different CMWGs with rectangular, triangular and sine teeth. (c, d) Simulation results of the transmission spectra (c) and group delay responses (d) for different width variations Δ*w* = 200, 400, and 600 nm. (e, f) Simulation results of the transmission spectra (e) and group delay responses (f) for different corrugation depths *δ*_0_ = 150, 250, and 350 nm. (g, h) Simulation results of the transmission spectra (g) and group delay responses (h) for different apodization length ratios *η* = 0, 0.025, 0.05, and 0.1. (i) Simulation results of GDR as apodization length ratio *η* varies. (j, k) Schematic diagram (j) and simulation results of light propagation (k) for the mode multiplexer.

Figure 2g,h shows the simulation results with different apodization length ratios *η* of 0, 0.025, 0.05 and 0.1. As the apodization ratio *η* is increased, a slight narrowing of the bandwidth is observed, whereas the GDR is simultaneously well‐suppressed. Figure 2i presents the GDR as a function of the apodization length ratio *η*, the GDR exhibits a rapid reduction when *η* is increased from 0 to 0.01. The GDR subsequently reaches a minimum value of approximately ± 0.48 ps at an apodization ratio *η* of approximately 0.10. Further increasing the ratio results in a slight rebound in the GDR magnitude. Consequently, the optimal apodization ratio is determined to be 0.10. Regarding the mode (de)multiplexer, its design principle relying on the adiabatic evolution of supermodes, has been clearly discussed in our previous work [31]. The optimized parameters for each segment in Figure 2j are (*w*_a1_, *w*_b1_) = (1.3, 0.8) μm and (*w*_a2_, *w*_b2_) = (1.7, 0.3) μm. The gap widths (*w*_g1_, *w*_g2_, *w*_g3_) = (1.5, 0.25, 1.5) μm and the taper lengths (*L*_1_, *L*_2_, *L*_3_) = (75, 150, 75) μm are selected to ensure adequate efficiency and the simulation result of light propagation at 1550 nm is shown in Figure 2k. Finally, the optimized parameters are determined as center width *w* = 1.7 μm, width variation Δ*w* = 400 nm, rectangular teeth with corrugation depth *δ*_0_ = 250 nm, and apodization length ratio *η* = 0.10. Under these conditions, the simulated bandwidth and dispersion of a single CMWG are 27.5 nm and 1.74 ps/nm, respectively.

As previously established, the DTDC employs an architecture comprising *N* stages of cascaded CMWGs integrated with (*N* + 1) 2 × 2 MZSs. Here, the number *N* is set as 5, and this yields a binary‐weighted distribution of CMWGs across the five stages: 1, 2, 4, 8, 16, respectively. This configuration results in a total of *M* = 2^5^ − 1 = 31 CMWGs. Given that the simulated dispersion of a single CMWG is 1.74 ps/nm, the total dispersion can be tuned from 0 to 53.94 ps/nm with a minimum step of 1.74 ps/nm. The simulated spectra of the designed DTDC, incorporating estimated process‐condition losses of 0.2, 0.25, and 0.3 dB/cm, are shown in Figure 3a–c. These results clearly illustrate that longer wavelengths in the bandwidth with longer propagation distances consequently incur higher loss. It is important to note that the overall device loss is directly tied to the platform's propagation loss. Considering that state‐of‐the‐art silicon nitride platforms have demonstrated propagation losses as low as 0.014 dB/cm [32], the total loss of the DTDC can be reduced remarkably by adopting advanced fabrication technologies. Figure 3d illustrates the accumulated dispersion increases linearly as the number of the CMWGs passed through increases. To evaluate the impact of the fabrication non‐uniformity on the device performance, a numerical analysis is performed by introducing a certain random perturbation, as shown in Figure 3e,f. For simplicity, the cumulative random phase errors are modeled as a unified normal distribution of grating period deviations, with a mean of 0 and a standard deviation *σ* of 0.6 nm. The numerical results indicate that the operational bandwidth and the insertion loss are nearly unchanged, attributed to the lowered index contrast of silicon nitride, thus showing superior fabrication tolerance than SOI CMWGs [33]. Regarding the dispersion, the random errors definitely introduce additional GDRs. For a single CMWG, the GDR increases from ± 0.48 to ± 1.02 ps. As the number of CMWGs increases to for example 31, the GDR increases from ± 14.88 to ± 16.18 ps. As a result, advanced fabrication with low random phase errors is still crucial for achieving low GDRs. Besides, the MZSs integrated into the DTDC utilize broadened phase shifters. Following validated design guidelines from our previous work [34], this specific design ensures low phase errors, corresponding to lower power consumption for active tuning.

**FIGURE 3:**
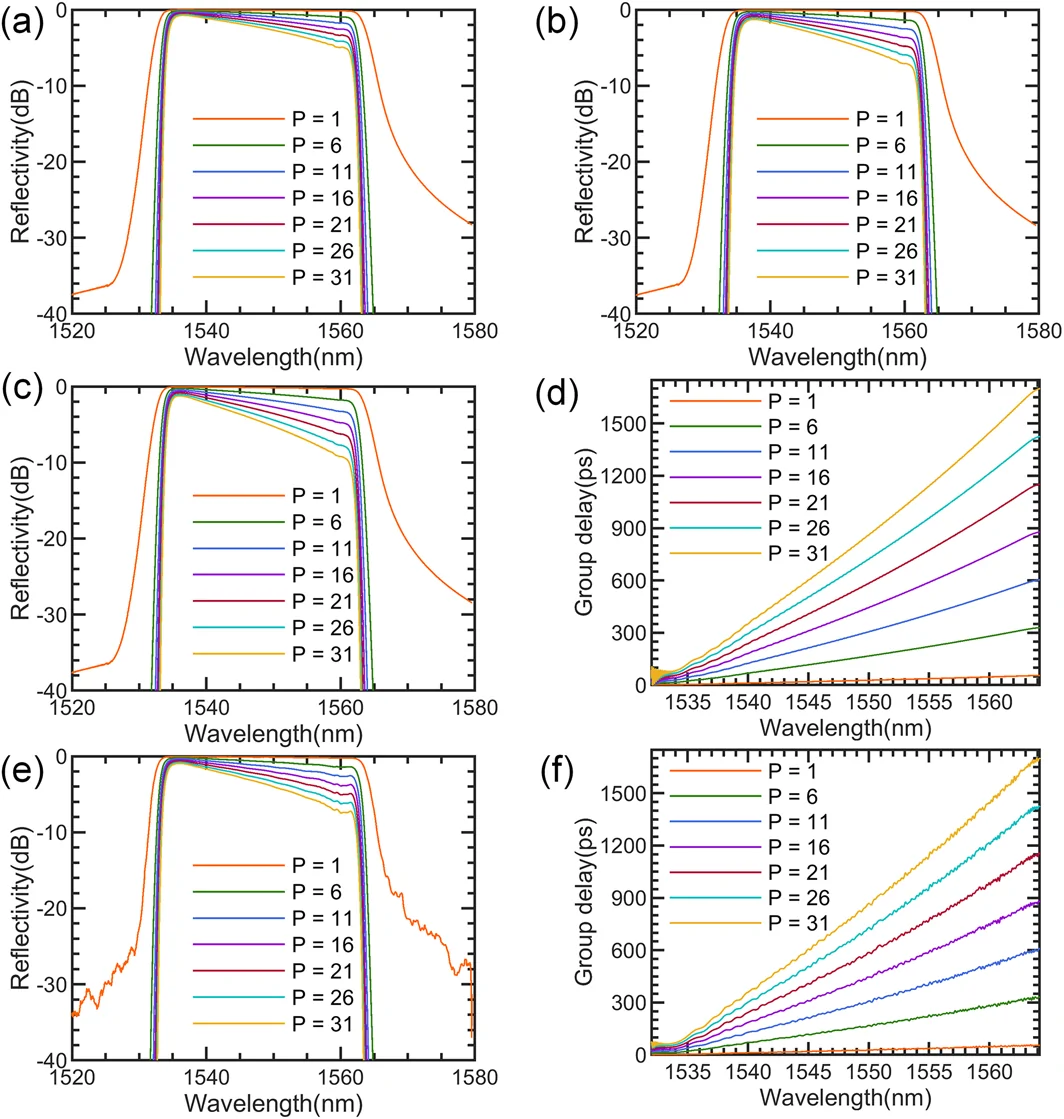
Simulated transmission spectra for the CMWGs with different propagation losses of (a) 0.15 dB/cm, (b) 0.25 dB/cm, and (c) 0.35 dB/cm. (d) Simulated group delay for the CMWGs with a propagation loss of 0.25 dB/cm. (e) Simulated transmission spectra for the CMWGs with random phase errors. (f) Simulated group delay for the CMWGs with random phase errors.

## 3 Fabrication and Measurement

The designed DTDC was fabricated on a silicon nitride‐on‐insulator wafer with a 400‐nm‐thick silicon nitride layer and a buried‐oxide layer by Shanghai Industrial μTechnology Research Institute (SITRI) via a standardized 8‐inch foundry process compatible with high‐volume manufacturing. The waveguide patterns were first defined by DUV lithography and subsequently transferred via inductively coupled plasma (ICP) dry‐etching. A top cladding oxide layer was deposited via the plasma enhanced chemical vapor deposition (PECVD) process. Then the metal heaters were deposited on the upper cladding layer via evaporation. The mask layout and microscopic images of the fabricated chip are shown in Figure 4a,b. The footprint of the whole five‐stage DTDC is approximately 7.4 × 0.7 mm^2^. The core region for the cascaded CMWGs covers a small area of 4.3 × 0.3 mm^2^. Note that the spacing between adjacent CMWGs is chosen as 10 μm to be safe for decoupling, and it is possible to be reduced further to be denser.

**FIGURE 4:**
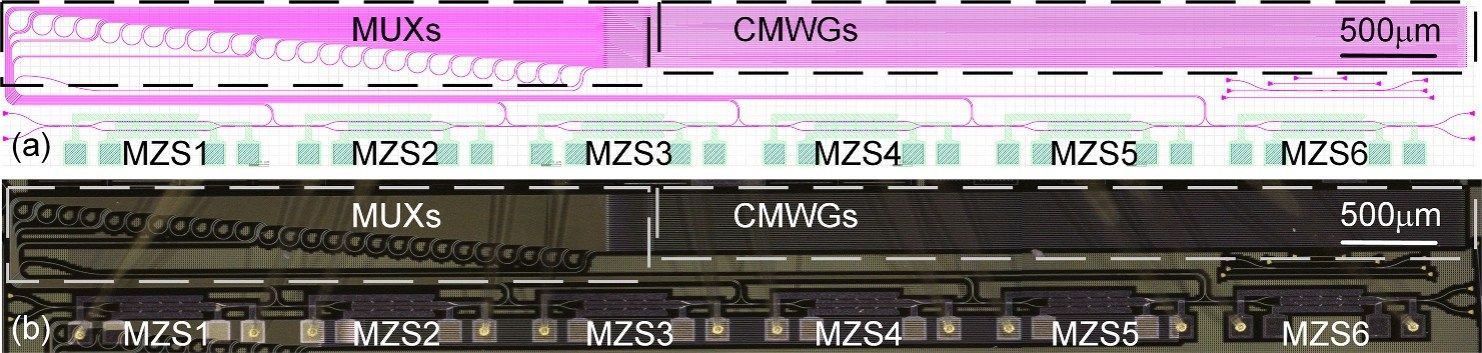
(a) Mask layout of the designed DTDC; (b) microscopic images of the fabricated DTDC.

The switching states of the MZSs, essential for digitally tuning the dispersion, were regulated by multichannel voltage sources. To measure the optical transmission performance, a broadband amplified spontaneous emission (ASE) light source was injected into the chip and the output spectrum was recorded with an optical spectrum analyzer (OSA, Yokogawa AQ6370C). The group delay measurements were performed with the dedicated experimental setup schematically illustrated in Figure 5a. A single‐wavelength optical signal, sourced from a tunable laser (Santec TSL‐570), was fed into an optical intensity modulator (IM). A radio frequency (RF) signal from a synthesized clock generator (SCG, SHF 78124A) was simultaneously applied to the IM to generate a sinusoidal optical signal. Polarization controllers (PCs) were strategically placed to ensure the optimal polarization state of the optical signals, while an erbium‐doped fiber amplifier (EDFA) was employed to compensate for system optical loss before detection. The output signals were then directed into a digital communication analyzer (DCA, Keysight 86100D) for precise acquisition of the time‐domain waveform and subsequent group delay calculation.

**FIGURE 5:**
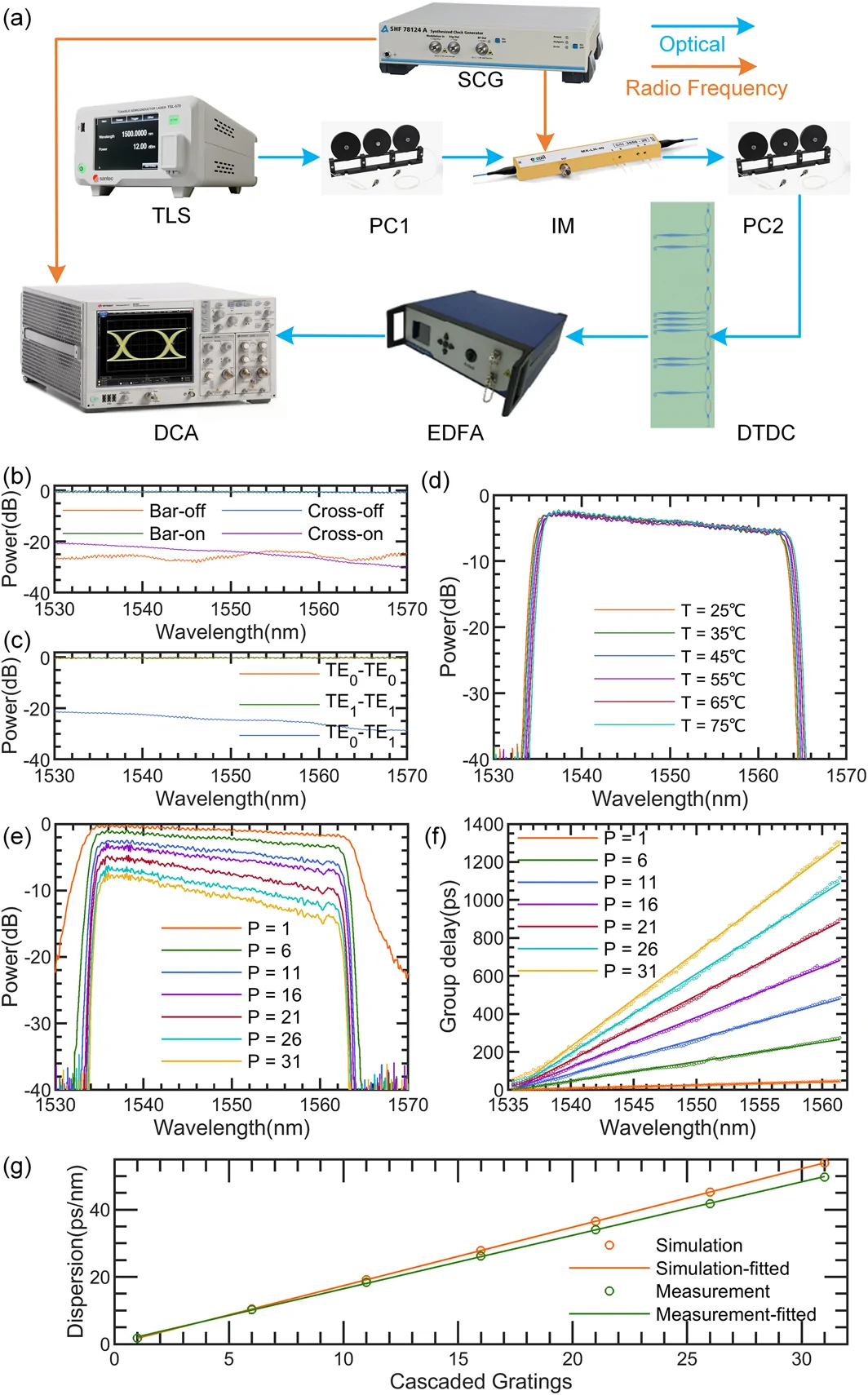
(a) Experimental setup for group delay measurements. DCA, digital communication analyzer; EDFA, erbium‐doped fiber amplifier; IM, intensity modulator; PC, polarization controller; SCG, synthesized clock generator; TLS, tunable laser source. (b, c) Transmission measurement results of the MZS (b) and the mode (de)multiplexer (c). (d) Transmission measurement results of 10 CMWGs at different temperatures *T* of 25°C, 35°C, 45°C, 55°C, 65°C, 75°C. (e, f) Measurement results of transmission (e) and group delay responses (f) of different numbers of CMWGs passed through. (g) Simulation and measurement results of dispersion with different numbers of CMWGs passed through.

Figure 5b displays the measured transmission spectra of the optimized MZS featuring the broadened phase shifter design. Characterization confirms that the MZS exhibits an ultra‐low excess loss and a high extinction ratio of > 20 dB. For these six MZSs, their operating voltages to achieve π‐phase shift are 3.98, 3.99, 3.99, 3.95, 3.97 and 4.02 V, respectively, demonstrating excellent consistency and low phase errors for them. Correspondingly, the power consumptions for these six MZSs are 199, 199, 199, 198, 199, and 203 mW, respectively, and the average power consumption (*P*_0_) is 199.5 mW for an MZS. The operational energy efficiency of the present DTDC can be evaluated through its digital switching logic. Since the power consumption of an MZS in its cross state is virtually negligible, the total power consumption for any specific tuning level is determined easily by the number *a* of the MZSs switched on to the bar state, and the maximal power consumption is given as 6*P*_0_ when all MZSs are on.

The measured transmission spectra of a pair of mode (de)multiplexers are presented in Figure 5c. The results demonstrate an ultra‐low excess loss and a low crosstalk of < −20 dB, critical to the circulator‐free characteristic of the overall DTDC architecture. Figure 5d shows the measured transmission spectra of 10 CMWGs at different temperatures *T* of 25°C, 35°C, 45°C, 55°C, 65°C, 75°C. As the temperature increases, the refractive index slightly rises and the bandwidth shifts towards longer wavelengths, moving by approximately 0.2 nm per 10°C rise, demonstrating much higher thermal stability than SOI platform.

The transmission spectra corresponding to different numbers of active CMWGs within the DTDC are displayed in Figure 5e. It can be seen that the loss of the DTDC increases progressively and the operational bandwidth undergoes a slight narrowing as the number of utilized CMWGs increases. The operational bandwidth of the fabricated DTDC is approximately 26 nm, spanning from 1535.4 to 1561.4 nm. For the fully engaged configuration of 31 CMWGs, the losses of the longest and shortest wavelengths within the operational bandwidth are 14.01 and 8.55 dB respectively, which is attributed to varying propagation distances (about 24.8 cm for the longest wavelength), indicating a low propagation loss of 0.22 dB/cm. The tilted spectral response could potentially be equalized by tailoring the corrugation depth of the gratings to deliberately reduce the reflectivity of wavelengths that travel shorter distances, thereby balancing the differential propagation loss accumulated over varying optical paths, which will be considered carefully in the future work. This propagation loss is significantly lower than previous work on SOI [25], which is about 1.3 dB/cm and the propagation loss can be further reduced when more advanced fabrication technologies are adopted. The measured group delay responses for various numbers of active CMWGs are shown in Figure 5f. The results clearly demonstrate that the group delay increases progressively across the operational bandwidth. Furthermore, Figure 5g illustrates the direct consequence of dispersion accumulation: the resultant dispersion increases linearly as a function of the total number of CMWGs utilized. For the fully engaged configuration of 31 CMWGs, the device achieves a maximal dispersion of 49.72 ps/nm and a maximal group delay up to 1304 ps, corresponding to an average dispersion of 1.60 ps/nm and an average group delay of 42.06 ps for a single CMWG, which represent the fundamental digital tuning step precision of the controller. The corresponding GDR of the fully engaged configuration of 31 CMWGs is ± 14.70 ps, accounting for only approximately 1.14% of the total delay, significantly outperforming previous work on SOI and thin‐film lithium niobate [26, 28].

## 4 Conclusion

In this work, we successfully develop a DTDC using low‐loss silicon nitride multimode waveguide gratings for on‐chip dispersion management. This architecture is realized by integrating a cascade of *N* stages of CMWGs and (*N* + 1) 2 × 2 MZSs. The DTDC enables precise digital manipulation of dispersion over a wide range, specifically from 0 to (2*^N^* − 1) *D*_0_, with a minimum tuning resolution of *D*_0_. A key architectural advantage is the use of mode (de)multiplexers integrated with the CMWGs, which effectively eliminates the need for external circulators, thereby enhancing design flexibility and integration density. To validate the concept, a device with *N* = 5 stages, utilizing 31 CMWGs and six MZSs, has been successfully designed and fabricated via a standardized 8‐inch foundry process compatible with high‐volume manufacturing for the first time, which ensures cost‐effective mass production and paves the way for large‐scale application. Each CMWG provides a dispersion *D*_0_ of approximately 1.60 ps/nm across a 26‐nm‐wide bandwidth, with a low propagation loss of 0.22 dB/cm and a low GDR of about 1.14% of the total delay. By appropriately configuring the MZSs, a maximum accumulated dispersion of 49.72 ps/nm is achieved. This maximum dispersion corresponds to a maximum group delay as high as 1304 ps. Although the present device is demonstrated for the TE polarization, achieving polarization‐independent operation is desired for some practical deployment in optical fiber systems. This could be addressed in the future work by adopting thicker silicon nitride photonic waveguides with low birefringence [35] or by introducing polarization‐diversity circuits [36, 37]. Overall, the present DTDC offers high flexibility for on‐chip dispersion management, combining a broad operational bandwidth with fine, digitally controlled tuning steps. The design is inherently scalable and the silicon nitride platform ensures low‐loss property, allowing for the extension of the number of stages to achieve substantially high dispersion. The high thermal stability and cost‐effectiveness due to the wafer‐scale fabrication also pave a robust scalable routine for practical applications. The demonstrated DTDC is highly attractive and poised to become an essential component for advanced dispersion management in critical optical systems, such as microwave photonics and long‐haul high‐speed optical communications.

## Author Contributions

**Weihan Wang:** conceptualization, investigation, writing – original draft, methodology, validation, visualization, writing – review and editing, formal analysis, data curation. **Ruitao Ma** **:** conceptualization, investigation, methodology, validation, software, formal analysis, writing – review and editing. **Shujun Liu** **:** conceptualization, methodology, writing – review and editing. **Mingyu Zhu** **:** methodology. **Zian Cao** **:** investigation. **Zexu Wang** **:** visualization. **Weike Zhao** **:** writing – review and editing. **Yiwei Xie** **:** funding acquisition. **Zejie Yu** **:** funding acquisition. **Daoxin Dai** **:** conceptualization, methodology, project administration, supervision, resources, funding acquisition, witing – original draft, writing – review and editing.

## Funding

This work was supported by the National Natural Science Foundation of China (Grants 62321166651, U23B2047, 62375238, and 62205292) and the Zhejiang Provincial Natural Science Foundation of China (Grant Nos LDT23F04012F05 and LD22F040004).

## Conflicts of Interest

The authors declare no conflicts of interest.

## Data Availability

The datasets generated and analyzed during the current study are available from the corresponding author upon reasonable request.
